# Community Health Workers and Precision Medicine: Results of a Randomized Clinical Trial on Patient Knowledge, Healthcare Use, and Evidence-Based Care

**DOI:** 10.3390/cancers18081247

**Published:** 2026-04-14

**Authors:** Emily H. Wood, Lesly Lopez Guzman, Jajaira L. Reynaga, Gerardo Villicana, Ysabel Duron, Lisa G. Rosas, Dale O’Brien, Zachary M. Koontz, Manali I. Patel

**Affiliations:** 1 Division of Oncology, Stanford University School of Medicine, Palo Alto, CA 94305, USA; ehwood@stanford.edu; 2 Milken Institute School of Public Health, George Washington University, Washington, DC 20052, USA; 3 Pacific Cancer Care, Monterey, CA 93940, USA; lguzman@pacificcancercare.com (L.L.G.); zkoontz@pacificcancercare.com (Z.M.K.); 4 Department of Psychology, University of California, Santa Cruz, CA 95064, USA; jlreynag@ucsc.edu; 5 Independent Researcher, Monterey, CA 93940, USA; 6 The Latino Cancer Institute, San Jose, CA 95112, USA; yduron@latinocancerinstitute.org; 7 Department of Epidemiology and Population Health, Stanford University, Stanford, CA 94305, USA; lgrosas@stanford.edu; 8 Department of Medicine, Stanford University, Palo Alto, CA 94303, USA; 9 Cancer Patients Alliance, Pacific Grove, CA 93950, USA; daleob@stanford.edu; 10 Veterans Affairs Palo Alto Health Care System, Palo Alto, CA 94304, USA

**Keywords:** precision medicine, community health worker, tumor testing, genomic testing, molecular profiling, health equity, biomarker testing

## Abstract

**Background/Objectives:** Molecular testing and targeted therapeutics remain inequitably delivered among patients with cancer. In response, we refined a multilevel intervention directed at clinicians, payers, and patients to determine whether it could improve patient knowledge and receipt of precision cancer care more than an intervention directed at clinicians and payers alone. **Methods:** This patient-level randomized trial was conducted in a community oncology clinic among low income and racial and ethnic minority adults who were newly diagnosed with cancer or relapsed disease. We compared a two-level enhanced usual care intervention, in which patients received usual care, clinicians received annual precision medicine training, and payers eliminated prior authorization for molecular testing and targeted therapeutics (control group), with a three-level intervention, in which patients received enhanced usual care along with a patient-level 12 month precision medicine education component led by community health workers (intervention group). The primary outcome was precision medicine knowledge. Secondary outcomes were health-related quality of life (HRQOL), patient activation, satisfaction, acute care use, molecular testing, and targeted treatment. **Results:** Among 110 participants, the three-level intervention resulted in greater improvement in knowledge of precision medicine compared with the two-level control group (mean difference of 4.17, 95% CI of 2.33–7.48; *p* < 0.001). Intervention participants also had greater patient activation and satisfaction with care, fewer emergency department visits and hospitalizations, and greater receipt of molecular testing and targeted therapy compared with patients in the control group. **Conclusions:** Multilevel interventions that include patient-level education can improve care delivery gaps. **Trial Registration:** clinicaltrials.gov, NCT04843332.

## Simple Summary

This randomized clinical trial tested whether a three-level intervention comprising clinician training, payer prior authorization elimination, and patient education and support led by a community health worker was more effective than a two-level intervention comprising clinician training and payer authorization elimination in improving patient knowledge regarding precision medicine over time. Participants randomized to the three-level intervention had greater improvements in knowledge about precision medicine, were more activated and satisfied, had greater precision medicine testing and treatment receipt, and had lower hospitalizations and ER visits than those randomized to the two-level intervention. These findings support three-level interventions targeting patient, clinician, and payer levels to improve cancer care.

## 1. Introduction

Precision medicine in cancer care refers to the use of tumor-specific biomarkers to guide individualized treatment decisions. This approach often relies on molecular testing, which involves the analysis of tumor DNA, RNA, or proteins to identify biomarkers that inform diagnosis, prognosis, and treatment selection [1,2]. Genomic testing is a subset of molecular testing that focuses on specific alterations in tumor DNA such as mutations, which may help predict response to therapy [3] and guide the use of targeted therapies treatments designed to inhibit specific molecular alterations or signaling pathways that drive tumor growth and progression [4]. National guidelines recommend molecular testing for cancers that can be treated with targeted therapeutics to optimize treatment, improve quality of life, and improve survival [5,6,7,8,9,10,11]. However, gaps remain in the routine delivery of molecular testing and corresponding targeted treatments [12], especially for low-income, uninsured, Medicaid insured, and racial and ethnic minority patients with cancer in the US [10,12,13,14,15,16,17,18,19,20,21]. Furthermore, inequitable access to precision cancer remains a global concern given that most precision medicine research and treatment infrastructure are concentrated in high-income countries [22,23,24]. These gaps have important consequences for patients as molecular testing is required to determine whether guideline-recommended targeted therapies can be provided. The lack of such testing results in poorer clinical outcomes and quality of life.

In our prior work, clinicians, payers, and patients identified that limited patient and clinician knowledge, changing guidelines, and prior authorization challenges prohibited routine molecular testing and evidence-based treatment delivery [15,25,26,27,28,29]. Together, these findings highlight the need for interventions that can simultaneously address barriers at multiple levels of the healthcare system. In response, we adapted a prior effective multilevel intervention [30] in collaboration with a 15-member Community Advisory Board (CAB) comprising patients, caregivers, community-based organizations, public health officials, clinicians, payers, and clinic and hospital executives [31]. The co-designed intervention included: (1) annual clinician training; (2) prior authorization elimination for molecular testing and targeted treatment; and (3) bilingual community health worker (CHW)-led patient education and support.

Prior studies have identified multilevel barriers to the delivery of precision cancer care, including limited patient and clinician knowledge structural barriers, and payer-related barriers. However, most existing interventions target a single level (e.g., clinician-level or patient-level) and there is limited research on coordinated, multilevel strategies that address patient, clinician, and payer barriers simultaneously. In particular, it is unclear whether multilevel interventions that include clinician-level, payer-level, and patient-level education and support can improve patient knowledge of precision medicine and downstream care delivery outcomes among diverse populations.

To address this gap, we conducted this randomized clinical trial to determine whether a three-level intervention comprising clinician training, prior authorization elimination, and patient education improved patient knowledge of precision medicine (primary outcome) more than a two-level enhanced usual care intervention comprising clinician training and prior authorization elimination alone. Secondarily, we evaluated the effects on patient-reported outcomes, acute care use, molecular testing, and targeted treatment. Thus, by testing a multilevel intervention using a randomized design, this study aimed to generate evidence on scalable strategies to improve patient knowledge, engagement, and routine delivery of precision cancer care.

## 2. Methods

### 2.1. Study Design and Oversight

In collaboration with a community-based oncology clinic in Monterey County, California, we enrolled patient participants between 3 May 2021 and 31 October 2023. The study was a parallel-group, patient-level, randomized controlled trial with a 1:1 allocation ratio. The trial was designed as a superiority study to evaluate whether the three-level intervention improved outcomes compared with the two-level enhanced usual care arm, as a means to generate gold standard evidence to inform practice and state-based policy. The development, patient-level component design, and protocol, described previously and summarized below [31,32], were guided by an active community advisory board that met quarterly throughout the entire study, initially in-person, then virtually during the COVID-19 pandemic. Community partner organizations led by co-investigators (YD, DO) collaborated on the study conception and the State of California grant proposal, which funded this work. The co-designed protocol (Appendix A) was approved by the research review committee at the clinic and the Stanford University Institutional Review Board.

#### 2.1.1. Participant Selection

Eligibility included patients 18 years of age or older newly diagnosed with a solid or hematologic malignancy or with progression or recurrence identified by imaging or biopsy and confirmed by an oncologist. All participants were required to have capacity to verbally consent in English or Spanish. All cancer types and stages were eligible and individuals were eligible if they self-identified as a racial or ethnic minority or were insured by either Medicaid (Medi-Cal) or employment with a local agricultural company, or were uninsured or had a household income of at least 80% or less of the area median income.

#### 2.1.2. Procedures and Randomization

An English–Spanish bilingual research assistant screened for eligibility using the electronic health record, verified eligibility with the potential participant either in-person or by telephone, and obtained verbal consent. A statistician randomly assigned all consenting participants, stratified by stage of disease, using a computer-generated sequence, within 1 week of consent, to one of two groups: three-level intervention comprising enhanced usual care with patient education and support (intervention group) or two-level enhanced usual care alone comprising usual care along with annual clinician training and prior authorization elimination (control group). Block randomization was used (block size 2, 4, or 6 within each stratum) and stratified by stage and cancer type. The randomization schema was uploaded to REDCap 16.1.3 allowing the computer to assign new patients as they enrolled to one of the two study groups. The principal investigator (MIP), statistician, data abstractors, data outcomes assessors, and research assistants were blinded to randomization assignments; participants and community health workers were not blinded. Data analysis was conducted between 31 October 2024, and 15 February 2025. The principal investigator (MIP) conducted two site visit fidelity checks annually during the study to monitor adherence to protocol activities and, along with the local site investigator (ZK), adverse events.

#### 2.1.3. Interventions

***Addressing Latinx CANcer Care Equity (Intervention group)***

Participants randomized to the intervention group received enhanced usual care (described below) along with patient-level education and a support component led by one of six English–Spanish bilingual CHWs who engaged participants in 30-min semi-structured weekly telephone-based or in-person discussions for 4 months post-enrollment and monthly thereafter for 12 months post-enrollment or until death, whichever was first. CHWs discussed molecular testing, targeted treatments, general cancer topics, goals of care, and integrated plain language handouts and animated videos in English and Spanish, co-developed by the research team with the CAB into discussions. CHWs were provided with a standardized semi-structured script covering the key content for each sessions. CHWs also encouraged participants to maintain a notebook with questions for their clinical teams and assisted participants in preparing questions—including those specifically regarding precision medicine. The purpose was to enhance patients’ skills and confidence for engaging with their clinical teams during and between clinic appointments. CHWs also screened for and referred participants to community-based resources for health-related social needs. All CHWs were trained using a standardized adapted curricula developed by PI (MIP) and refined by the CAB on motivational interviewing, navigation, palliative care, cancer basics, molecular testing, targeted therapies, and research procedures (described in a prior publication [32]).

***Enhanced Usual Care (Control group)***

All participants received usual care, which included outpatient care delivered by oncology clinicians, nurses, nutritionists, and palliative specialists. In addition, the local site investigator (ZK) trained all clinic oncologists annually on molecular testing and targeted therapeutic guidelines and the local payer organizations eliminated prior authorization for molecular testing and corresponding targeted therapeutics. Usual care varied by diagnosis and treatment plan but typically involved an initial visit after a diagnosis of cancer to discuss treatments, and follow-up visits every 4–8 weeks to monitor treatment progress.

#### 2.1.4. Study Outcomes

The primary outcome was change in knowledge of precision medicine between groups over time. Secondary outcomes included changes in patient-reported outcomes (i.e., health-related quality of life, patient activation, satisfaction with decision, prognosis and treatment preferences) and differences in acute care use, molecular testing, and targeted therapy within 12 months follow-up between groups.

Primary and secondary patient-reported outcomes were measured by validated assessments that were either self-administered or administered by a research staff member (if a participant needed assistance) in English or Spanish. All assessments were administered at baseline (time of enrollment) and 3, 6, and 12 months post-enrollment (described below).

***Knowledge of precision medicine (Primary Outcome)***

Knowledge of precision medicine was measured using a 7-item multiple-choice survey adapted from Davies et al. [33], which assessed molecular testing and treatment knowledge. The survey is scored from 0 to 100% with one correct answer choice for each question and higher scores indicating greater knowledge. The CAB advised that a clinically meaningful difference was equivalent to 3 correct responses (score of 43%) [34].

***Secondary Outcomes***

***Health-Related Quality of Life (HRQOL)***

HRQOL was measured using the validated 27-item Functional Assessment of Cancer Therapy–General (Version 4) [35,36], which assessed 4 domains. Scores range from 0 to 108 with higher scores indicating greater HRQOL.

***Patient Activation***

Patient activation was measured using the validated 10-item Patient Activation Measure (PAM-10) [37,38]. Scores range from 0 to 100 to assess the degree to which patients have confidence and knowledge and proactively take actions to maintain and improve health. Higher scores indicate greater patient activation.

***Satisfaction with Decision***

Satisfaction with decision was measured using the validated 6-item Satisfaction with Decision [39], with scores that range from 6 to 30 measuring the degree to which patients were satisfied with their medical decisions, with higher scores indicating greater satisfaction.

***Prognosis and Treatment Preference***

Prognosis and treatment preference was assessed using a 4-item prognosis and treatment preference questionnaire, adapted from Weeks et al. (1998) [40], and descriptively reported.

***Acute Care Use***

A research team member abstracted the use and dates of all emergency department visits and hospitalizations, molecular testing, and, if applicable, targeted treatment receipt using the electronic health record between the baseline and 12 months post-enrollment or death, whichever was first. The National Comprehensive Cancer Network guidelines were used at the start of the study to define cancers for which molecular testing and germline testing was recommended, with a focus on advanced cancers of all histologies including non-small cell lung cancer, colon and rectal, breast, ovarian, fallopian tube, primary peritoneal, prostate, pancreatic, biliary tract, gastric, esophageal, bladder, melanoma, endometrial, and tumor agnostic (all solid tumor) metastatic or advanced cancers.

**Demographic and Clinical Characteristics**

Demographic variables were self-reported at the time of enrollment and included age, gender, ethnicity, race, educational attainment, marital status, household income and size, primary language spoken at home, English proficiency, health insurance type, employment status and birthplace. Cancer health literacy was measured at baseline using the validated Cancer Health Literacy Test-6, a 6-item assessment with scores based on the number of correct answers, with higher scores indicating greater health literacy [41].

A research assistant abstracted baseline clinical characteristics through an electronic health record review, which included primary cancer diagnosis, cancer stage, recurrence, the Charlson Comorbidity Index [42], and the Eastern Cooperative Oncology Group Performance Status [43].

### 2.2. Statistical Analyses

All statistical tests were conducted using Stata Version 16 [44]. We estimated that a sample size of 110 would provide greater than 90% power at a 2-sided alpha of 0.05 to detect a clinically meaningful increase in the mean knowledge score from a baseline of 14% (equivalent to a mean of 1 question out of 7) to 43% (equivalent to a mean of 3 correct responses of 7) over time, assuming a 12% loss of follow-up based on prior data [33]. All primary analyses were analyzed on an intention-to-treat basis (including all available outcome data up to the time of death or withdrawal) and adjusted for age, gender, ethnicity, race, education, cancer diagnosis, and cancer stage.

#### Primary and Secondary Analyses

The expected mean difference in change in patient knowledge of precision medicine was estimated by generalized estimating equation (GEE) models modeled as a function of randomized group, categorical time (baseline, 3 months, 6 months, 12 months), and an interaction term between the treatment group and time, with an exchangeable correlation clustered within person and adjusted for age, gender, ethnicity, race, education, cancer diagnosis, and cancer stage. Significance was assessed between groups over time using a type III F-test on the interaction term. Expected mean differences in HRQOL, patient activation, and satisfaction with decision were modeled as per the primary analyses. Prognosis and treatment preference responses were reported descriptively. For dichotomous secondary outcomes, odds ratios (ORs) and 95% confidence intervals (CIs) were calculated using GEE-log binomial models and for continuous acute care use outcomes, incidence rate ratios (IRRs) and 95% CIs were calculated using negative binomial models with an offset term for the log-transformed length of follow-up and adjusted as per primary analyses. Secondary outcomes were prespecified and considered exploratory. Therefore, no adjustment was made for multiple comparisons. Effect estimates and 95% confidence intervals are presented to facilitate interpretation of the magnitude and consistency of findings.

## 3. Results

**Figure 1:**
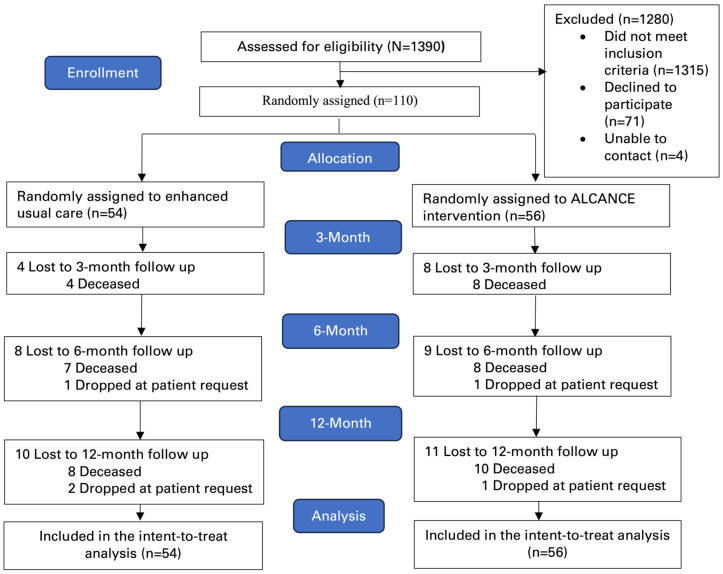
Enrollment and randomization of participants.

Of 1390 patients screened for eligibility, 110 were eligible and randomized. A total of 18 (16.4%) died and 3 (2.7%) withdrew consent prior to the 12 month follow-up (Figure 1). Table 1 depicts demographic and clinical characteristics. The median age was 59 years (range 21–87); 58 (52.7%) were female; 89 (80.9%) were Hispanic or Latino; 8 (7.3%) were Asian or Pacific Islander; 3 (2.7%) were Native Hawaiian or Pacific Islander; 10 (9.1%) were Non-Hispanic Black; 26 (24.1%) were Non-Hispanic White; and 13 (11.8%) identified as having multiple races. Fifty (45.5%) selected race as “not listed’, all of whom used the prompted write-in field to identify as “Hispanic,” “Latino,” or “Mexican.” A total of 75 (68.2%) attained less than or the equivalent to a high school diploma or General Education Development (GED); 60 (54.6%) were married; 48 (43.6%) had annual household incomes less than $49,999 USD; 78 (66.4%) had households comprising more than one individual; 68 (61.8%) spoke Spanish as their preferred language; 51 (46.4%) were MediCal or other public organization insured; 39 (35.5%) were privately insured; and 28 (25.5%) were Medicare insured. Mean cancer health literacy was low (mean score (SD) 3.27 (1.09)). Gastrointestinal cancer was the most common cancer type among 40 (36.4%) of the participants. A total of 83 (75.5%)) were diagnosed with stage 3 or 4 cancer and 51 (46.4%) had an Eastern Cooperative Oncology Group Performance Status of 1. There appeared to be no differences in the demographic and clinical characteristics between groups.

### 3.1. Primary Outcome (Figure 2, Appendix Table A1)

**Figure 2:**
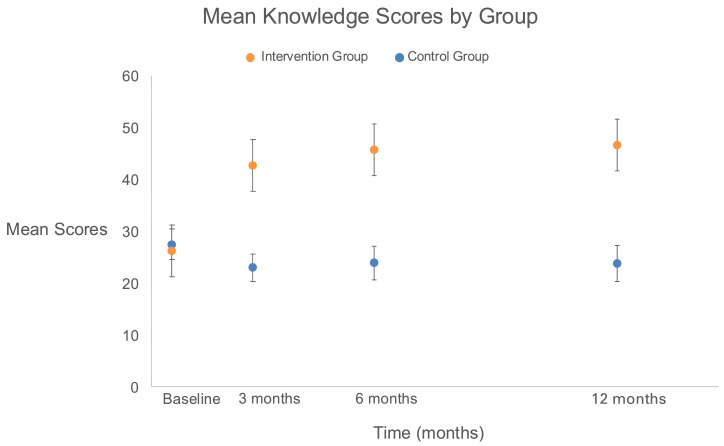
Mean precision knowledge scores by group at 3, 6, and 12 Months. All participants in both control (54, 100.0%) and treatment (56, 100.0%) groups completed the knowledge assessment items at baseline. A total of 50 participants (92.6%) in the control group and 48 (85.7%) in the intervention group completed the knowledge assessment questions at 3 months after study enrollment. Missingness was only observed because of death among 4 (7.4%) participants in the control group and 8 (14.3%) participants in the intervention group before this 3 month assessment. A total of 46 participants (85.2%) in the control group and 47 (83.9%) in the intervention group completed the knowledge assessment questions at 6 months after study enrollment. Missingness was observed because of death (7, 13.0%) and dropout (1, 1.9%) for participants in the control group and death (8, 14.3%) and dropout (1, 1.8%) for participants in the intervention group before this 6 month assessment. A total of 44 participants (81.5%) in the control group and 45 (80.4%) in the intervention group completed the knowledge assessment questions at 12 months after study enrollment. Missingness was observed because of death (8, 14.8%) and dropout (2, 3.7%) for participants in the control group and due to death (10, 17.9%) and dropout (1, 1.8%) for participants in the intervention group before this 12 month assessment. Effect estimates were expected mean differences in knowledge scores between groups from baseline. Compared with the control group, the intervention group had a greater increase in mean scores between groups from baseline to 3 months (3.31 [95% CI, 1.88 to 5.81; *p* < 0.001]), 6 months (3.82 [95% CI, 2.16 to 6.78; *p* < 0.001]), and 12 months (4.17 [95% CI, 2.33 to 7.48; *p* < 0.001]).

Participants had low baseline knowledge of precision medicine with approximately one question correct out of seven (14%) across both groups (mean score (standard deviation (SD)): intervention group with 1.57 (1.18) versus control group with 1.65 (1.29)) (Figure 2, Appendix Table A1). Intervention group participants had a greater increase in mean knowledge scores (SD) at each assessment period with mean differences, in correct questions between groups, of 3.31 ([95% CI, 1.88 to 5.81, *p* < 0.001) between baseline and 3 months, 3.82 ([95% CI, 2.16–6.78]; *p* < 0.001) between baseline and 6 months, and 4.17 ([95% CI, 2.33 to 7.48]; *p* < 0.001) between baseline and 12 months.

### 3.2. Secondary Outcomes

#### 3.2.1. HRQOL (Figure 3a, Appendix Table A1)

**Figure 3:**
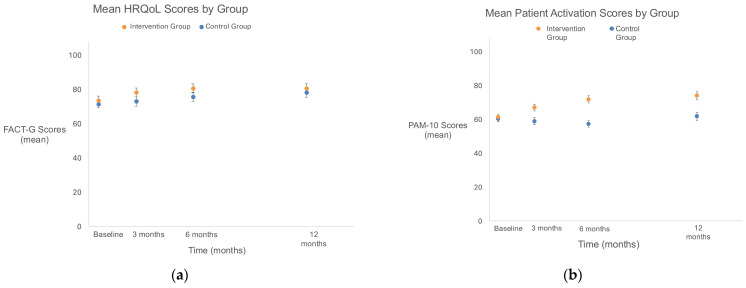
(**a**) Legend: Mean HRQoL by group measured via the FACT-G. A total of 50 participants (92.6%) in the control group and 48 (85.7%) in the intervention group completed this survey question at 3 months after study enrollment. Missingness was only observed because of death among 4 (7.4%) participants in the control group and 8 (14.3%) participants in the intervention group before this 3 month assessment. A total of 46 participants (85.2%) in the control group and 47 (83.9%) in the intervention group completed this survey question at 6 months after study enrollment. Missingness was observed because of death (7, 13.0%) and dropout (1, 1.9%) for participants in the control group and death (8, 14.3%) and dropout (1, 1.8%) for participants in the intervention group before this 6 month assessment. A total of 44 participants (81.5%) in the control group and 45 (80.4%) in the intervention group completed this survey question at 12 months after study enrollment. Missingness was observed because of death (8, 14.8%) and dropout (2, 3.7%) for participants in the control group and due to death (10, 17.9%) and dropout (1, 1.8%) for participants in the intervention group before this 12 month assessment. Effect estimates were expected mean differences in HRQoL scores between groups from baseline. Compared with the control group, the intervention group had no statistically significant difference in mean scores between groups from baseline to 3 months (4.02 [95% CI, −1.52 to 9.75]; *p* = 0.159), 6 months (4.11 [95% CI, −1.53 to 9.75]; *p* = 0.156), and 12 months (1.90 [95% CI,−3.82 to 7.62]; *p* = 0.522). FACT-G, Functional Assessment of Cancer Therapy—General; HRQoL, health-related quality of life. (**b**) Legend: Mean patient activation by group. A total of 50 participants (92.6%) in the control group and 48 (85.7%) in the intervention group completed this survey question at 3 months after study enrollment. Missingness was only observed because of death among 4 (7.4%) participants in the control group and 8 (14.3%) participants in the intervention group before this 3 month assessment. A total of 46 participants (85.2%) in the control group and 47 (83.9%) in the intervention group completed this survey question at 6 months after study enrollment. Missingness was observed because of death (7, 13.0%) and dropout (1, 1.9%) for participants in the control group and death (8, 14.3%) and dropout (1, 1.8%) for participants in the intervention group before this 6 month assessment. A total of 44 participants (81.5%) in the control group and 45 (80.4%) in the intervention group completed this survey question at 12 months after study enrollment. Missingness was observed because of death (8, 14.8%) and dropout (2, 3.7%) for participants in the control group and due to death (10, 17.9%) and dropout (1, 1.8%) for participants in the intervention group before this 12 month assessment. Effect estimates were expected mean differences in Patient Activation Measure scores between groups from baseline. Compared with the control group, the intervention group had a greater increase in mean scores between groups from baseline to 3 months (7.43 [95% CI, 0.90 to 13.97]; *p* = 0.026), 6 months (14.29 [95% CI, 7.63 to 20.94]; *p* < 0.001), and 12 months (12.10 [95% CI, 5.35 to 18.85]; *p* < 0.001). PAM-10, Patient Activation Measure-10.

HRQOL was similar at baseline across both groups (mean score (SD): intervention with 73.73 (18.21) versus control group with 71.60 (17.05)) (Figure 3a, Appendix Table A1). Mean scores increased over time within both groups with no statistically significant difference between groups.

#### 3.2.2. Patient Activation (Figure 3b, Appendix Table A1)

Patient activation was similar at baseline across both groups (mean score (SD): intervention group with 61.36 (13.82) versus control group with 60.50 (13.28)). Intervention group participants had greater increases in mean scores over time than control group participants with a mean difference of 7.43 ([95% CI, 0.90 to 13.97]; *p* < 0.001) between baseline and 3 months, 14.29 ([95% CI, 7.63 to 20.94]; *p* < 0.001) between baseline and 6 months, and 12.10 ([95% CI, 5.35 to 18.85]; *p* < 0.001) between baseline and 12 months (Figure 3b, Appendix Table A1).

#### 3.2.3. Satisfaction with Decision (Appendix Table A1, Appendix Figure A1)

**Figure A1:**
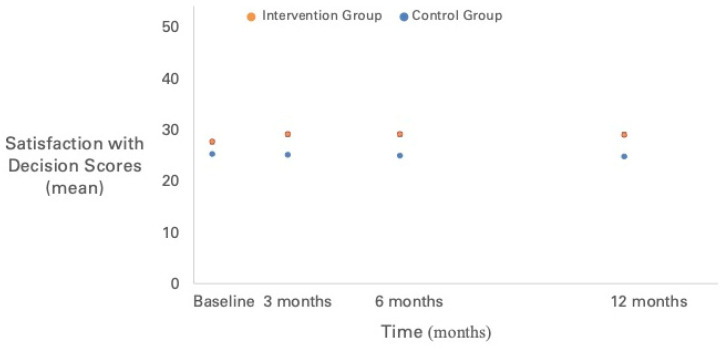
Mean satisfaction with decision by group. Mean satisfaction with decision by group over time. Scores range from 6 to 30 with higher scores indicating greater satisfaction. A total of 48 (85.7%) in the intervention group and 50 participants (92.6%) in the control group completed this survey question at 3 months after study enrollment. Missingness was only observed because of death among 8 (14.3%) participants in the intervention group and 4 (7.4%) participants in the control group before this 3 month assessment. A total of 47 (83.9%) in the intervention group and 46 participants (85.2%) in the control group completed this survey question at 6 months after study enrollment. Missingness was observed because of death (8, 14.3%) and dropout (1, 1.8%) for participants in the intervention group and death (7, 13.0%) and dropout (1, 1.9%) for participants in the control group before this 6 month assessment. A total of 45 (80.4%) in the intervention group and 44 participants (81.5%) in the control group completed this survey question at 12 months after study enrollment. Missingness was observed because of death (10, 17.9%) and dropout (1, 1.8%) for participants in the intervention group and due to death (8, 14.8%) and dropout (2, 3.7%) for participants in the control group before this 12 month assessment. Effect estimates were expected mean differences in satisfaction with decision scores between groups from baseline. Compared with the control group, the intervention group had a greater increase in mean scores between groups from baseline to 3 months of 1.49 ([95% CI, 0.14 to 2.84]; *p* < 0.001), 6 months (1.46 [95% CI, 0.11 to 2.80]; *p* < 0.001), and 12 months (1.53 [95% CI, 0.17 to 2.89]; *p* < 0.001) between baseline and 12 months.

Mean (SD) satisfaction with decision scores were slightly higher among the intervention group (27.64 (3.32)) than the control group (25.27 (4.82)) at baseline (Appendix Table A1, Appendix Figure A1). Intervention group participants had greater increases in mean satisfaction scores with a mean difference of 1.49 ([95% CI, 0.14 to 2.84]; *p* < 0.001) between baseline and 3 months, 1.46 ([95% CI, 0.11 to 2.80]; *p* < 0.001) between baseline and 6 months, and 1.53 ([95% CI, 0.17 to 2.89]; *p* < 0.001) between baseline and 12 months.

#### 3.2.4. Prognosis and Treatment Preference (Appendix Table A2)

At baseline, a high proportion of participants across both groups believed their cancer was curable, did not know how long most patients with their disease lived, and believed the goal of treatment was to extend life. Over time, across both groups, more participants believed their cancer was incurable (Appendix Table A2).

#### 3.2.5. Acute Care Use (Appendix Table A3)

Between baseline and 12 months, 12 (21.4%) intervention group participants and 32 (59.3%) control group participants had at least one emergency department visit. Intervention group participants had lower odds of emergency department use compared with control group participants (OR, 0.18 [95% CI, 0.08 to 0.43]; *p* < 0.001) (Appendix Table A3). Between baseline and 12 months, 10 (17.9%) intervention group participants and 25 (46.3%) control group participants had a least one hospitalization. Intervention group participants had lower odds of hospitalization use compared with control group participants (OR, 0.25 [95% CI, 0.11 to 0.60]; *p* = 0.01). The intervention group also had lower frequencies of both emergency department (IRR, 0.53 [95% CI, 0.37 to 0.76]; *p* = 0.001) and hospitalization use (IRR, 0.45 [95% CI, 0.30 to 0.96]; *p* = 0.002) than the control group.

#### 3.2.6. Molecular Testing and Receipt of Targeted Treatment (Figure 4)

**Figure 4:**
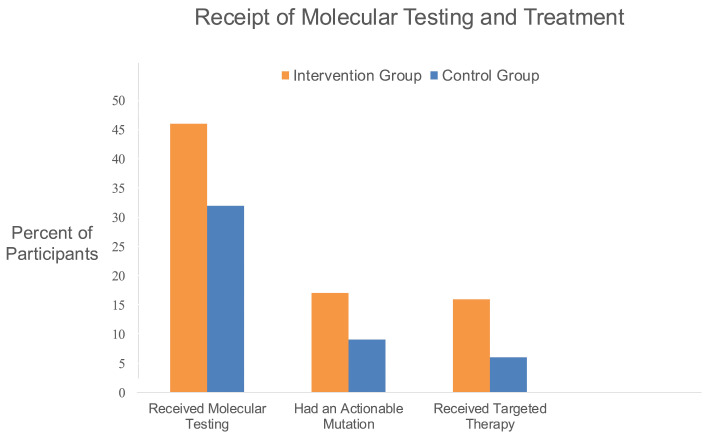
Molecular testing and treatment receipt by group. Electronic health record abstraction was conducted to assess proportion with receipt of precision medicine testing and targeted treatment between baseline to 12 months post-enrollment (or death or dropout). At 12 month follow-up, the intervention group had higher odds of both precision medicine (molecular) testing (OR, 3.16 [95% CI, 1.32 to 7.57]; *p* = 0.008) and receipt of targeted therapy (OR, 3.20 [95% CI 1.14 to 8.94]; *p* = 0.02). There were no statistically significant differences in receipt of targeted therapy for the 26 participants across both groups who received molecular testing and who had a targetable or actionable mutation (intervention group with 16 (94%) versus control group with 6 (66.7%); OR, 8.02 [95% CI 0.69 to 92.71]).

At 12 months follow-up, more intervention group participants (46 (82.1%)) than control group participants (32 (59.3%)) had received molecular testing (Figure 4) with 3-fold higher odds (OR, 3.16 [95% CI, 1.32 to 7.57]; *p* = 0.008). Among those who received molecular testing, there were no statistically significant differences between groups in the proportion with an actionable mutation (intervention group with 17 (36.9%) versus control group with 9 (28.2%)). Overall, intervention group participants had 3-fold higher odds of receiving targeted therapy (OR, 3.20 [95% CI, 1.14 to 8.94]; *p* = 0.02). However, among the 26 participants across both groups who received testing and had an actionable mutation, there were no statistically significant differences in the receipt of targeted therapy (intervention group with 16 (94%) versus control group with 6 (66.7%); *p* = 0.07; OR 8.02 [95% CI, 0.69 to 92.70).

## 4. Discussion

In this single-site, randomized clinical trial, a three-level intervention comprising clinician training, payer elimination of prior authorization, and patient-level education and support increased patient knowledge of precision medicine more than a two-level intervention comprising clinician training and payer elimination of prior authorization alone. In exploratory secondary analyses, the intervention also improved patient activation and satisfaction, reduced emergency department and hospital use, and improved molecular testing and targeted therapy receipt.

While the three-level intervention resulted in statistically significant improvements in knowledge, patient activation, and several care delivery outcomes, its clinical significance requires careful consideration. Notably, there was no statistically significant improvement in health-related quality of life. This may reflect the relatively short follow-up period and the advanced disease status of many participants, in whom changes in quality of life are more difficult to detect. It is also possible that improvements in knowledge represent an important intermediate outcome that may translate into patient-reported outcome benefits over a longer time horizon.

Despite limited precision cancer care delivery among low-income, publicly insured or uninsured, and racial and ethnic minority populations, few targeted multilevel interventions exist [16,17,19,45,46,47,48,49,50]. As patient knowledge and activation are key determinants of clinical outcomes [51,52,53,54], it is not surprising that tailored patient education interventions such as ours, among others [55,56,57,58,59,60], show benefit. Here, the addition of the patient-level component was more effective than the clinician- and payer-level interventions alone, and, as shown in our prior work evaluating such interventions in other aspects of cancer care delivery, improved patient activation and satisfaction [56,61]. Such interventions address previously cited barriers to routine evidence-based cancer care delivery [15,25]. This positive impact may reflect improved patient communication with clinicians, leading to greater knowledge and receipt of evidence-based care [62,63,64,65]. However, as this was not measured directly, it is important to note that there are several mechanisms that could explain the observed effects, including increased patient knowledge, improved confidence in engaging with clinicians, and enhanced care through community health worker support. While community health workers may have facilitated education, communication, and linkage to resources, the relative contribution of each of these components cannot be determined from the current data. Future studies are needed to evaluate the specific pathways through which these multilevel interventions influenced patient outcomes.

Notably, our findings highlight the sheer importance of molecular testing as a basic but foundational component for evidence-based cancer treatment. Specifically, molecular testing and the receipt of targeted therapy was greater overall in the intervention group, yet when compared among those who received molecular testing across both groups, there were no differences in the receipt of the corresponding targeted therapeutics. While the three-level intervention was more effective across primary and multiple secondary endpoints, the clinician and payer-level interventions designed and implemented by our community and payer partners were foundational to addressing known underlying barriers, such as unconscious biases, knowledge gaps, and time pressures, that prevented routine molecular testing and the subsequent delivery of targeted therapeutics [19,66,67]. Our approach involved additional clinician training; however, interventions that limit clinician reliance also include default orders in the electronic health record, automated patient dashboards, and electronic health record-embedded decision support tools [68]. Furthermore, policies such as state Medicaid expansion and support for community health workers, in addition to prior authorization elimination, can further mitigate administrative and logistical burdens of molecular testing. Such support is crucial to the intervention’s feasibility and sustainability across settings.

This study has limitations. First, despite diverse participants, generalizability may be limited due to the focus on one geographic region and a single community oncology practice. Findings may not apply well to other settings. Second, contamination could have occurred in the control group, as CHWs infrequently may have interacted with patients in the control group. While it was an uncommon occurrence, patient logs indicate that community health workers occasionally served as interpreters in participants’ clinical visits. Such contamination could, however, bias findings toward the null indicating an even greater effect of the three-level intervention. Third, while we attempted to maintain blinding, interactions between community health workers and clinicians to discuss patient care needs were possible. Fourth, there was limited missing data due to death, and, to a lesser extent, participant withdrawal. However, there were no meaningful differences in death or withdrawal by group. Finally, multiple secondary outcomes were evaluated without adjustment for multiple comparisons, which increases the risk of type I error and a priori were designated as exploratory outcomes. Strengths of the study include our collaborative design with a community advisory board and inclusion of payer and employer organizations to enhance the intervention effect and real-world adoption and sustainability. This co-designed approach has resulted in the continuation of the three-level intervention as part of usual care in the clinic and county, serving as an important intervention that could be replicated in other areas nationally.

## 5. Conclusions

Patient-level education and support enhance delivery of precision cancer care for low-income and racial and ethnic minorities with cancer.

## Figures and Tables

**Table 1:** Baseline demographic and clinical characteristics of study participants.

Variable	Total (*N* = 110)	Intervention Group (*n* = 56)	Control Group (*n* = 54)
Age in years—median (range)	59 (21–87)	59 (26–86)	59 (21–87)
Gender—no. (%)			
Female	58 (52.7)	24 (47.4)	28 (58.5)
Male	52 (47.3)	32 (52.6)	26 (41.5)
Ethnicity—no. (%)			
Hispanic	89 (80.9)	43 (76.8)	46 (85.2)
Race (%)—no. (%)			
Asian or Pacific Islander	8 (7.3)	5 (8.9)	3 (5.6)
Native Hawaiian or Pacific Islander	3 (2.7)	1 (1.8)	2 (3.7)
Non-Hispanic Black	10 (9.1)	6 (10.7)	4 (7.4)
Non-Hispanic White	26 (24.1)	15 (26.8)	11 (20.4)
Multiple races	13 (11.8)	4 (7.1)	9 (16.7)
Race not listed ^a^	50 (45.5)	25 (44.6)	25 (46.3)
Education—no. (%)			
Less than high school	53 (48.2)	22 (39.3)	31 (57.4)
High school or GED	22 (20.0)	14 (25.0)	8 (14.8)
Some college	16 (14.5)	10 (17.9)	6 (11.1)
Associate degree (2-year)	10 (9.1)	6 (10.7)	4 (7.4)
College graduate	6 (5.5)	4 (7.1)	2 (3.7)
Master’s degree	3 (2.7)	0 (0.0)	3 (5.6)
Marital status—no. (%)			
Married	60 (54.6)	31 (55.4)	29 (53.7)
Divorced	12 (10.9)	10 (17.9)	2 (3.7)
Widowed	9 (8.2)	2 (3.6)	7 (13.0)
Separated	5 (4.6)	4 (7.1)	1 (1.9)
Never married	15 (13.6)	6 (10.7)	9 (16.7)
Member of unmarried couple	8 (7.3)	3 (5.4)	5 (9.3)
Prefer not to answer	1 (0.9)	0 (0.0)	1 (1.9)
Annual household income (US Dollars)—no. (%)			
<$24,999	20 (26.4)	15 (26.8)	14 (25.9)
$25,000–≤$34,999	16 (14.6)	11 (19.6)	5 (9.3)
$35,000–≤$49,999	12 (10.9)	5 (8.9)	7 (13.0)
$50,000–≤$74,999	9 (8.2)	8 (14.3)	1 (1.9)
$75,000–≤$100,000	6 (5.5)	5 (8.9)	1 (1.9)
$100,000–≤$149,999	4 (3.6)	0 (0.0)	4 (7.4)
≥$150,000	1 (0.9)	1 (1.8)	0 (0.0)
I do not know my annual household income	19 (17.3)	8 (14.3)	11 (20.4)
I prefer not to answer about my income	14 (12.7)	3 (5.4)	11 (20.4)
Have children—no. (%)	86 (78.2)	43 (76.8)	43 (79.6)
Household size—no. (%)			
Single	26 (23.6)	13 (23.2)	13 (24.1)
1	11 (13.1)	4 (7.1)	7 (13.0)
2	24 (28.6)	12 (21.4)	12 (22.2)
3	21 (25.0)	16 (28.6)	5 (9.3)
4+	28 (33.3)	11 (19.7)	17 (31.5)
Primary language at home— No. (%)			
English	39 (35.5)	25 (44.6)	14 (25.9)
Spanish	68 (61.8)	30 (53.6)	38 (70.4)
Other	3 (2.7)	1 (0.9)	2 (1.8)
English spoken as a 2nd language ^b^			
Very well	8 (7.3)	3 (5.4)	5 (9.25)
Well	11 (10)	4 (7.1)	7 (13.0)
Not very well	25 (22.7)	12 (21.4)	13 (24.1)
Not at all	26 (23.6)	12 (21.4)	14 (25.9)
Insurance type ^c^—no. (%)			
Private	39 (35.5)	21 (37.5)	18 (33.3)
Medi-Cal/public	51 (46.4)	28 (50.0)	23 (42.6)
Medicare	28 (25.5)	12 (21.4)	16 (29.6)
Private + public	1 (0.9)	0 (0.0)	1 (1.9)
Other insurance	8 (7.3)	2 (3.6)	6 (11.1)
Uninsured ^d^	2 (1.8)	1 (1.8)	1 (1.9)
Employment status—no. (%)			
Full-time	19 (17.3)	7 (12.5)	12 (22.2)
Part-time	5 (4.5)	4 (7.1)	1 (1.9)
Unemployed	16 (78.2)	8 (14.3)	8 (14.8)
Retired	20 (18.2)	8 (14.3)	12 (22.2)
Disabled	41 (37.3)	26 (46.4)	15 (27.8)
Other	9 (8.2)	3 (5.4)	6 (11.1)
Birthplace—no. (%)			
Born in the US	36 (32.7)	22 (39.3)	14 (25.9)
Born outside the US	73 (66.4)	34 (60.7)	39 (72.2)
Cancer health literacy ^e^—Mean (SD)	3.27 (1.09)	3.29 (1.17)	3.26 (1.03)
Declined to answer	1 (0.9)	0 (0.0)	1 (1.9)
Primary diagnosis—No. (%)			
Breast	26 (23.6)	17 (30.4)	9 (16.7)
GI	40 (36.4)	19 (33.9)	21 (38.9)
GU	20 (18.2)	9 (16.1)	11 (20.4)
Head/neck	3 (2.7)	2 (3.6)	1 (1.9)
Heme	10 (9.1)	4 (7.1)	6 (11.1)
Thoracic	5 (4.5)	2 (3.6)	3 (5.6)
Other	6 (5.5)	3 (5.4)	3 (5.6)
Stage—no. (%)			
1	11 (10.0)	7 (12.5)	4 (7.4)
2	16 (14.5)	12 (21.4)	4 (7.4)
3	34 (30.9)	15 (26.8)	19 (35.2)
4	49 (44.5)	22 (39.3)	27 (50.0)
Recurrent cancer—no. (%)	26 (23.6)	13 (23.2)	13 (24.1)
Charlson comorbidity index—mean ± SD	6.01 ± 3.02	6.1 ± 3.3	6.0 ± 2.9
Eastern Cooperative Oncology Group Performance Status ^f^—no. (%)			
0	49 (44.5)	21 (37.5)	28 (51.9)
1	51 (46.4)	29 (51.8)	22 (40.7)
2	7 (6.4)	4 (7.1)	3 (5.6)
3	3 (2.7)	2 (3.6)	1 (1.9)
4	0 (0)	0 (0)	0 (0)

^a^ All participants who selected “Race not listed” self-reported their race as Hispanic when prompted with a write-in field. ^b^ For those who reported speaking a language other than English as their primary language, a self-report item about how well they spoke English was collected. Those who reported speaking English as their primary language did not receive this item. One individual from the control group did not respond to this item. ^c^ Insurance is reported as a “check all that apply field”, thus categories will not sum to 100%. ^d^ Includes those who reported “pay in cash for healthcare services”. ^e^ Cancer health literacy was assessed using the Cancer Health Literacy Test-6 (CHLT-6), a six-item questionnaire with dichotomized as well as multiple-choice questions designed to screen and identify an individual’s cancer health literacy level. Scores are based on the number of correct answers with higher scores indicating greater cancer health literacy. A score of ≥5 indicates adequate cancer health literacy. ^f^ Performance status was assessed using the Eastern Cooperative Oncology Group Performance Status measure, a 6-point scale from 0 to 5 that described a cancer patient’s ability to carry out daily activities. 0 represents fully active with no restrictions and 5 indicates death.

## Data Availability

Data will not be shared. Reason: We did not receive IRB approval to share the data.

## Abbreviations

The following abbreviations are used in this manuscript:

ALCANCE	Addressing Latinx Cancer Care Equity
CHW	Community Health Worker
PCC	Pacific Cancer Care
HRQOL	Health-Related Quality of Life
NGS	Next-Generation Sequencing

## Appendix A

**Table A1:** Precision medicine knowledge and patient-reported outcomes by group.

Assessment and Time Point	Intervention Group (*n* = 56) Mean (SD)	Control Group (*n* = 54) Mean (SD)	Expected Mean Difference (95% CI)	*p*
Knowledge ^a^				
Baseline	1.57 (1.18)	1.65 (1.29)	--------------------------------	-------
3 months	2.56 (1.79)	1.38 (1.10)	3.31 (1.88–5.81)	<0.001
6 months	2.75 (1.71)	1.43 (1.32)	3.82 (2.16–6.78)	<0.001
12 months	2.80 (1.73)	1.43 (1.39)	4.17 (2.33–7.48)	<0.001
Health-Related Quality of Life ^b^				
Baseline	73.73 (18.21)	71.60 (17.05)	--------------------------------	------------
3 months	78.35 (20.23)	73.14 (19.82)	4.02 (−1.52–9.75)	0.16
6 months	80.81 (19.54)	75.70 (19.13)	4.11 (−1.53–9.75)	0.16
12 months	80.73 (20.92)	78.36 (20.23)	1.90 (−3.82–7.62)	0.52
Patient Activation ^c^				
Baseline	61.36 (13.82)	60.50 (13.28)	--------------------------------	------------
3 months	67.07 (14.21)	58.98 (15.38)	7.43 (0.90–13.97)	*p* < 0.001
6 months	71.96 (14.88)	57.48 (12.48)	14.29 (7.63–20.94)	*p* < 0.001
12 months	74.13 (16.51)	61.91 (15.85)	12.10 (5.35–18.85)	*p* < 0.001
Satisfaction with Decision ^d^				
Baseline	27.64 (3.32)	25.27 (4.82)	--------------------------------	-----------
3 months	29.09 (2.61)	25.14 (5.56)	1.49 (0.14–2.84)	0.03
6 months	29.13 (3.02)	25.00 (4.94)	1.46 (0.11–2.80)	0.03
12 months	29.02 (3.03)	24.79 (6.23)	1.53 (0.17–2.89)	0.02

Abbreviations: SD, standard deviation; CI, confidence interval. ^a^ Knowledge of precision medicine was assessed using a 7-item multiple-choice questionnaire regarding molecular testing and treatment knowledge. Scores range from 0 to 100% with one correct answer choice for each question and higher scores indicating greater knowledge. All participants in both intervention (56, 100.0%) and control (54, 100.0%) groups completed the knowledge assessment items at baseline. A total of 48 (85.7%) in the intervention group and 50 participants (92.6%) in the control group completed the knowledge assessment questions at 3 months after study enrollment. Missingness was only observed because of death among 8 (14.3%) participants in the intervention group and 4 (7.4%) participants in the control group before this 3 month assessment. A total of 47 (83.9%) in the intervention group and 46 participants (85.2%) in the control group completed the knowledge assessment questions at 6 months after study enrollment. Missingness was observed because of death (8, 14.3%) and dropout (1, 1.8%) for participants in the intervention group and death (7, 13.0%) and dropout (1, 1.9%) for participants in the control group before this 6 month assessment. A total of 45 (80.4%) in the intervention group and 44 participants (81.5%) in the control group completed the knowledge assessment questions at 12 months after study enrollment. Missingness was observed because of death (10, 17.9%) and dropout (1, 1.8%) for participants in the intervention group and due to death (8, 14.8%) and dropout (2, 3.7%) for participants in the control group before this 12 month assessment. Effect estimates were expected mean differences in knowledge scores between groups from baseline. ^b^ Health-related quality of life by group was assessed using the validated Functional Assessment of Cancer Therapy—General (FACT-G), a 27-item questionnaire where scores range from 0 to 108 and higher scores indicate greater health-related quality of life. A total of 48 (85.7%) in the intervention group and 50 participants (92.6%) in the control group completed this survey question at 3 months after study enrollment. Missingness was only observed because of death among 8 (14.3%) participants in the intervention group and 4 (7.4%) participants in the control group before this 3 month assessment. A total of 47 (83.9%) in the intervention group and 46 participants (85.2%) in the control group completed this survey question at 6 months after study enrollment. Missingness was observed because of death (8, 14.3%) and dropout (1, 1.8%) for participants in the intervention group and death (7, 13.0%) and dropout (1, 1.9%) for participants in the control group before this 6 month assessment. A total of 45 (80.4%) in the intervention group and 44 participants (81.5%) in the control group completed this survey question at 12 months after study enrollment. Missingness was observed because of death (10, 17.9%) and dropout (1, 1.8%) for participants in the intervention group and due to death (8, 14.8%) and dropout (2, 3.7%) for participants in the control group before this 12 month assessment. Effect estimates were expected mean differences in HRQoL scores between groups from baseline. ^c^ Patient activation was measured using the Patient Activation Measure (PAM-10). Scores range from 0 to 100 with higher scores indicating greater activation. A total of 48 (85.7%) in the intervention group and 50 participants (92.6%) in the control group completed this survey question at 3 months after study enrollment. Missingness was only observed because of death among 8 (14.3%) participants in the intervention group and due to death among 4 (7.4%) participants in the control group before this 3 month assessment. A total of 47 (83.9%) in the intervention group and 46 participants (85.2%) in the control group completed this survey question at 6 months after study enrollment. Missingness was observed because of death (8, 14.3%) and dropout (1, 1.8%) for participants in the intervention group and due to death (7, 13.0%) and dropout (1, 1.9%) for participants in the control group before this 6 month assessment. A total of 45 (80.4%) participants in the intervention group and 44 participants (81.5%) in the control group completed this survey question at 12 months after study enrollment. Missingness was observed because of death (10, 17.9%) and dropout (1, 1.8%) for participants in the intervention group and due to death (8, 14.8%) and dropout (2, 3.7%) for participants in the control group before this 12 month assessment. Effect estimates were expected mean differences in scores between groups from baseline. ^d^ Satisfaction with decision was assessed using the validated Satisfaction with Decision measure. Scores range from 6 to 30 with higher scores indicating greater patient satisfaction with their medical decisions. A total of 48 (85.7%) in the intervention group and 50 participants (92.6%) in the control group completed this survey question at 3 months after study enrollment. Missingness was only observed because of death among 8 (14.3%) participants in the intervention group and among 4 (7.4%) participants in the control group before this 3 month assessment. A total of 47 (83.9%) in the intervention group and 46 participants (85.2%) in the control group completed this survey question at 6 months after study enrollment. Missingness was observed because of death (8, 14.3%) and dropout (1, 1.8%) for participants in the intervention group and due to death (7, 13.0%) and dropout (1, 1.9%) for participants in the control group before this 6 month assessment. A total of 45 (80.4%) in the intervention group and 44 participants (81.5%) in the control group completed this survey question at 12 months after study enrollment. Missingness was observed because of death (10, 17.9%) and dropout (1, 1.8%) for participants in the intervention group and due to death (8, 14.8%) and dropout (2, 3.7%) for participants in the control group before this 12 month assessment. Effect estimates were expected mean differences in Satisfaction with Decision scores between groups from baseline.

**Table A2:** Prognosis and treatment preference by group from baseline to 12 months post-enrollment.

Question ^a^	Response Option	Intervention Group *n* (%) (*n* = 56)	Control Group *n* (%) (*n* = 54)
Baseline ^b^			
#1 Is your cancer curable?	Yes	32 (57.1)	32 (59.3)
3 months	No	3 (5.4)	7 (12.9)
6 months	I don’t know	21 (37.5)	15 (27.8)
#2 How long do most patients with your disease live on average?	Less than 6 months	1 (1.8)	1 (1.9)
Health-Related Quality of Life ^b^	6 months–2 years	1 (1.8)	3 (5.6)
Baseline	More than 2 years	12 (21.4)	5 (9.3)
3 months	I don’t know	42 (75.0)	45 (83.3)
#3 The goal of my cancer treatment is to: (select all that apply) ^c^	Cure my disease	38 (67.9)	31 (57.4)
12 months	Help me feel better	13 (23.2)	22 (40.7)
Patient Activation ^c^	Extend my life	25 (44.6)	30 (55.6)
Baseline	I don’t know	9 (16.1)	6 (11.1)
#4 If you had to make a choice at this time, would you prefer a course of treatment that focuses on extending life as much as possible, even if it means having more pain and discomfort, or would you want a plan of care that focuses on relieving pain and discomfort, even if that means not living as long?	Extend life as much as possible	34 (60.7)	27 (50.0)
6 months	Relieve pain or discomfort as much as possible	12 (21.4)	16 (29.6)
12 months	I don’t know	10 (17.9)	11 (20.4)
3 months ^d^			
#1 Is your cancer curable?	Yes	25 (44.6)	25 (46.3)
3 months	No	10 (17.9)	7 (13.0)
6 months	I don’t know	13 (23.2)	18 (33.3)
#2 How long do most patients with your disease live on average?	Less than 6 months	1 (1.8)	2 (3.7)
	6 months–2 years	1 (1.8)	3 (5.6)
	More than 2 years	14 (32.1)	9 (16.7)
	I don’t know	28 (50.0)	36 (66.7)
#3 The goal of my cancer treatment is to: (select all that apply) ^c^	Cure my disease	28 (50.0)	26 (48.1)
	Help me feel better	15 (26.8)	14 (25.9)
	Extend my life	27 (48.2)	23 (42.6)
	I don’t know	3 (5.4)	7 (13.0)
#4 If you had to make a choice at this time, would you prefer a course of treatment that focuses on extending life as much as possible, even if it means having more pain and discomfort, or would you want a plan of care that focuses on relieving pain and discomfort, even if that means not living as long?	Extend life as much as possible	28 (50.0)	21 (38.9)
	Relieve pain or discomfort as much as possible	13 (23.2)	16 (29.6)
	I don’t know	7 (12.5)	13 (24.1)
6 months ^e^			
#1 Is your cancer curable?	Yes	24 (52.9)	22 (40.7)
	No	9 (16.1)	5 (9.3)
	I don’t know	13 (23.2)	19 (35.2)
#2 How long do most patients with your disease live on average?	Less than 6 months	2 (3.6)	0 (0.0)
	6 months–2 years	2 (3.6)	0 (0.0)
	More than 2 years	14 (25.0)	9 (16.7)
	I don’t know	28 (50.0)	37 (68.5)
#3 The goal of my cancer treatment is to: (select all that apply) ^c^	Cure my disease	27 (48.2)	26 (48.1)
	Help me feel better	9 (16.1)	9 (16.7)
	Extend my life	21 (37.5)	19 (35.2)
	I don’t know	2 (3.6)	5 (9.3)
#4 If you had to make a choice at this time, would you prefer a course of treatment that focuses on extending life as much as possible, even if it means having more pain and discomfort, or would you want a plan of care that focuses on relieving pain and discomfort, even if that means not living as long?	Extend life as much as possible	28 (50.0)	24 (44.4)
	Relieve pain or discomfort as much as possible	10 (17.9)	11 (20.4)
	I don’t know	8 (14.3)	11 (20.4)
12 months ^f^			
#1 Is your cancer curable? (labeled as progtreat3b in output?)	Yes	14 (25.0)	21 (38.9)
	No	31 (55.4)	23 (42.6)
	I don’t know	0 (0)	0 (0)
#2 How long do most patients with your disease live on average? (labeled as progtreat3a in output?)	Less than 6 months	5 (8.9)	1 (1.9)
	6 months–2 years	1 (1.8)	2 (3.7)
	More than 2 years	14 (25.0)	12 (22.2)
	I don’t know	25 (44.6)	29 (53.7)
#3 The goal of my cancer treatment is to: (select all that apply) ^c^	Cure my disease	25 (44.6)	21 (38.9)
	Help me feel better	13 (23.2)	11 (20.4)
	Extend my life	22 (39.3)	18 (33.3)
	I don’t know	5 (8.9)	4 (7.4)
#4 If you had to make a choice at this time, would you prefer a course of treatment that focuses on extending life as much as possible, even if it means having more pain and discomfort, or would you want a plan of care that focuses on relieving pain and discomfort, even if that means not living as long?	Extend life as much as possible	27 (48.2)	22 (40.7)
	Relieve pain or discomfort as much as possible	12 (21.4)	11 (20.4)
	I don’t know	6 (10.7)	11 (20.4)

^a^ Prognosis and treatment preference was assessed using a 4-item multiple-choice prognosis and treatment preference questionnaire, adapted from Weeks et al. (1998 [40]). ^b^ A total of 56 participants (100%) in the intervention group and 54 (100.0%) in the control group completed all the Prognosis and Treatment Preference survey questions at baseline. ^c^ Prognosis and Treatment Preference Item #3 was a select all that apply question, and answers may not sum to 100%. ^d^ A total of 48 participants (85.7%) in the intervention group and 50 (92.6%) in the control group completed all the Prognosis and Treatment Preference survey questions at 3 months post-enrollment. ^e^ A total of 46 participants (82.1%) in the intervention group and 46 (85.2%) in the control group completed all prognosis and treatment preferences questions at 6 months after study enrollment. ^f^ A total of 45 participants (80.4%) in the intervention group and 44 (81.5%) in the control group completed all prognosis and treatment preferences questions at 12 months after study enrollment.

**Table A3:** Healthcare use by group from baseline to 12 months post-enrollment.

Variable	Total (*N* = 110)	Intervention Group (*n* = 56)	Control Group (*n* = 54)	OR or IRR (95% CI)	*p*
Any ED visit use, no. (%) ^a^	44 (40.0)	12 (21.4)	32 (59.3)	OR: 0.18 (0.08–0.43)	<0.001
Mean ED visits, mean, (SD) ^b^	1.20 (1.86)	0.84 (1.62)	1.57 (2.02)	IRR: 0.53 (0.37–0.76)	0.001
Any hospital visit, no. (%) ^a^	35 (31.8)	10 (17.9)	25 (46.3)	OR: 0.25 (0.11–0.60)	0.01
Mean hospital visits, mean (SD) ^b^	0.88 (1.36)	0.55 (1.16)	1.22 (1.48)	IRR: 0.45 (0.30–0.96)	0.002

Abbreviations: ED, emergency department; IRRs, incidence rate ratios; OR, odds ratio; SD, standard deviation. ^a^ Odds Ratios (ORs) for any ED use and any hospitalization use were estimated using logistic regression models adjusted for age, gender, ethnicity, education, cancer diagnosis, and cancer stage. ^b^ Incidence rate ratios (IRRs) were estimated using negative binomial models offset for follow-up time and adjusted for age, gender, ethnicity, race, education, cancer diagnosis, and cancer stage. All ratios are expressed as referent to the control group.

## References

[1] Minhinnick A., Santos-Gonzalez F., Wilson M., Lorgelly P. (2025). How is value defined in molecular testing in cancer? A scoping review. Appl. Health Econ. Health Policy.

[2] El-Deiry W.S., Goldberg R.M., Lenz H.J., Shields A.F., Gibney G.T., Tan A.R., Brown J., Eisenberg B., Heath E.I., Phuphanich S. (2019). The current state of molecular testing in the treatment of patients with solid tumors, 2019. CA Cancer J. Clin..

[3] American Cancer Society Medical and Editorial Content Team Cancer-Related Genomic Testing and Genetic Testing. https://www.cancer.org/cancer/understanding-cancer/genes-and-cancer/genomic-genetic-testing.html.

[4] Burkett M.S. (2024). Molecular testing and precision oncology: An overview. Nursing.

[5] Seyhan A.A. (2022). The Current State of Precision Medicine and Targeted-Cancer Therapies: Where Are We?. Drug Target Selection and Validation.

[6] Tippur A. (2023). AI-Powered Precision Oncology: Computational Insights Redefining Therapeutic Landscapes. DHR Proc..

[7] Hoeben A., Joosten E.A., van den Beuken-van Everdingen M.H. (2021). Personalized medicine: Recent progress in cancer therapy. Cancers.

[8] Gambardella V., Tarazona N., Cejalvo J.M., Lombardi P., Huerta M., Roselló S., Fleitas T., Roda D., Cervantes A. (2020). Personalized medicine: Recent progress in cancer therapy. Cancers.

[9] Yang S.-R., Schultheis A.M., Yu H., Mandelker D., Ladanyi M., Büttner R. (2022). Precision medicine in non-small cell lung cancer: Current applications and future directions. Semin. Cancer Biol..

[10] Curtin M., Dickerson S.S. (2022). Precision medicine testing and disparities in health care for individuals with non-small cell lung cancer: A narrative review. Proceedings of the Oncology Nursing Forum.

[11] Chakravarty D., Johnson A., Sklar J., Lindeman N.I., Moore K., Ganesan S., Lovly C.M., Perlmutter J., Gray S.W., Hwang J. (2022). Somatic Genomic Testing in Patients with Metastatic or Advanced Cancer: ASCO Provisional Clinical Opinion. J. Clin. Oncol..

[12] National Cancer Institute Cancer Disparities. https://www.cancer.gov/about-cancer/understanding/disparities.

[13] Marinac C.R., Ghobrial I.M., Birmann B.M., Soiffer J., Rebbeck T.R. (2020). Dissecting racial disparities in multiple myeloma. Blood Cancer J..

[14] Patel M.I., Lopez A.M., Blackstock W., Reeder-Hayes K., Moushey A., Phillips J., Tap W. (2020). Cancer disparities and health equity: A policy statement from the American Society of Clinical Oncology. J. Clin. Oncol..

[15] Robert N.J., Nwokeji E.D., Espirito J.L., Chen L., Karhade M., Evangelist M.C., Spira A.I., Neubauer M.A., Bullock S.A., Coleman R.L. (2021). Biomarker tissue journey among patients (pts) with untreated metastatic non-small cell lung cancer (mNSCLC) in the US Oncology Network community practices. J. Clin. Oncol..

[16] Ragavan M.V., Borno H.T. (2023). The costs and inequities of precision medicine for patients with prostate cancer: A call to action. Proceedings of the Urologic Oncology: Seminars and Original Investigations.

[17] Amaral Duarte F., Aguiar Junior P.N., Dienstmann R., Ferreira C.G. (2023). Precision medicine in Thoracic Oncology: Understanding disparities to tackle inequities in access. Expert Rev. Pharmacoecon. Outcomes Res..

[18] Puckrein G.A. (2021). Leading Health and Cancer Advocacy Groups Unite to Reduce Racial Disparities in Cancer Care.

[19] Winn R., Winkfield K., Mitchell E. (2023). Addressing disparities in cancer care and incorporating precision medicine for minority populations. J. Natl. Med. Assoc..

[20] Gamble C.R., Huang Y., Wright J.D., Hou J.Y. (2021). Precision medicine testing in ovarian cancer: The growing inequity between patients with commercial vs medicaid insurance. Gynecol. Oncol..

[21] Dutta R., Vallurupalli M., McVeigh Q., Huang F.W., Rebbeck T.R. (2023). Understanding inequities in precision oncology diagnostics. Nat. Cancer.

[22] Barragan-Carrillo R., Asirwa F.C., Dienstmann R., Pendhakar D., Ruiz-Garcia E. (2025). Global oncology: Tackling disparities and promoting innovations in low-and middle-income countries. Am. Soc. Clin. Oncol. Educ. Book.

[23] Girisha K.M., Moosa S. (2024). Genomic testing in low-and middle-income countries (LMIC). Eur. J. Hum. Genet..

[24] Drake T.M., Knight S.R., Harrison E.M., Søreide K. (2018). Global inequities in precision medicine and molecular cancer research. Front. Oncol..

[25] Bruno D.S., Hess L.M., Li X., Su E.W., Zhu Y.E., Patel M. (2021). Racial disparities in biomarker testing and clinical trial enrollment in non-small cell lung cancer (NSCLC). J. Clin. Oncol..

[26] Rodriguez G.M., Leach M., Osorio J., Villicana G., Koontz Z., Wood E.H., Duron Y., O’Brien D., Rosas L.G., Patel M.I. (2022). Exploring cancer care needs for Latinx adults: A qualitative evaluation. Support. Care Cancer.

[27] Centers for Disease Control and Prevention (2024). What Is Health Literacy?. https://www.cdc.gov/health-literacy/php/about/index.html.

[28] Holden C.E., Wheelwright S., Harle A., Wagland R. (2021). The role of health literacy in cancer care: A mixed studies systematic review. PLoS ONE.

[29] Durand M.A., Yen R.W., O’Malley A.J., Schubbe D., Politi M.C., Saunders C.H., Dhage S., Rosenkranz K., Margenthaler J., Tosteson A.N.A. (2021). What matters most: Randomized controlled trial of breast cancer surgery conversation aids across socioeconomic strata. Cancer.

[30] Patel M.I., Kapphahn K., Wood E., Coker T., Salava D., Riley A., Krajcinovic I. (2023). Effect of a Community Health Worker–Led Intervention Among Low-Income and Minoritized Patients with Cancer: A Randomized Clinical Trial. J. Clin. Oncol..

[31] Wood E.H., Leach M., Villicana G., Goldman Rosas L., Duron Y., O’Brien D.G., Koontz Z., Patel M.I. (2023). A Community-Engaged Process for Adapting a Proven Community Health Worker Model to Integrate Precision Cancer Care Delivery for Low-income Latinx Adults with Cancer. Health Promot. Pract..

[32] Rodriguez G.M., Wood E.H., Xiao L., Duron Y., O’Brien D., Koontz Z., Roas L.G., Patel M.I. (2022). Community health workers and precision medicine: A randomized controlled trial. Contemp. Clin. Trials.

[33] Davies G., Butow P., Napier C.E., Bartley N., Juraskova I., Meiser B., Ballinger M.L., Thomas D.M., Schlub T.E., Best M.C. (2020). Development of the Patient Activation Measure (PAM): Conceptualizing and measuring activation in patients and consumers. Transl. Oncol..

[34] Rodriguez G.M., Wood E.H., Leach M., Villicana G., Murillo A., Rosas L.G., Duron Y., O’Brien D.G., Koontz Z., Patel M.I. (2022). Addressing Latinx CANcer Care Equity (ALCANCE) randomized controlled trial: Precision medicine and community health workers. J. Clin. Oncol..

[35] Cella D.F., Tulsky D.S., Gray G., Sarafian B., Linn E., Bonomi A., Silberman M., Yellen S.B., Winicour P., Brannon J. (1993). The Functional Assessment of Cancer Therapy scale: Development and validation of the general measure. J. Clin. Oncol..

[36] Functional Assessment of Cancer Therapy—General. https://www.facit.org/measures/FACT-G.

[37] Patient Activation Measure. https://www.insigniahealth.com/pam/.

[38] Hibbard J.H., Mahoney E.R., Stockard J., Tusler M. (2005). Development and testing of a short form of the patient activation measure. Health Serv. Res..

[39] Holmes-Rovner M., Kroll J., Schmitt N., Rovner D.R., Breer M.L., Rothert M.L., Padonu G., Talarczyk G. (1996). Patient satisfaction with health care decisions: The satisfaction with decision scale. Med. Decis. Mak..

[40] Weeks J.C., Cook E.F., O’Day S.J., Peterson L.M., Wenger N., Reding D., Harrell F.E., Kussin P., Dawson N.V., Connors A.F. (1998). Relationship between cancer patients’ predictions of prognosis and their treatment preferences. JAMA.

[41] Dumenci L., Matsuyama R., Riddle D.L., Cartwright L.A., Perera R.A., Chung H., Siminoff L.A. (2014). Measurement of cancer health literacy and identification of patients with limited cancer health literacy. J. Health Commun..

[42] Charlson M.E., Pompei P., Ales K.L., MacKenzie C.R. (1987). A new method of classifying prognostic comorbidity in longitudinal studies: Development and validation. J. Chronic Dis..

[43] Oken M.M., Creech R.H., Tormey D.C., Horton J., Davis T.E., McFadden E.T., Carbone P.P. (1982). Toxicity and response criteria of the Eastern Cooperative Oncology Group. Am. J. Clin. Oncol..

[44] StataCorp (2019). Stata Statistical Software: Release 16.

[45] Wilkerson A.D., Gentle C.K., Ortega C., Al-Hilli Z. (2024). Disparities in breast Cancer Care—How factors related to Prevention, diagnosis, and Treatment Drive Inequity. Healthcare.

[46] Islami F., Guerra C.E., Minihan A., Yabroff K.R., Fedewa S.A., Sloan K., Wiedt T.L., Thomson B., Siegel R.L., Nargis N. (2022). American Cancer Society’s report on the status of cancer disparities in the United States, 2021. CA Cancer J. Clin..

[47] Leech M.M., Weiss J.E., Markey C., Loehrer A.P. (2022). Influence of race, insurance, rurality, and socioeconomic status on equity of lung and colorectal cancer care. Ann. Surg. Oncol..

[48] Alio A.P., Wharton M.J., Fiscella K. (2022). Structural racism and inequities in access to Medicaid-funded quality cancer care in the United States. JAMA Netw. Open.

[49] Diaz A., Pawlik T.M. (2021). Geographic disparities in oncologic treatment and outcomes: The urban–rural divide. Ann. Surg. Oncol..

[50] Zahnd W.E., Murphy C., Knoll M., Benavidez G.A., Day K.R., Ranganathan R., Luke P., Zgodic A., Shi K., Merrell M.A. (2021). The intersection of rural residence and minority race/ethnicity in cancer disparities in the United States. Int. J. Environ. Res. Public Health.

[51] Hibbard J.H., Mahoney E., Sonet E. (2017). Does patient activation level affect the cancer patient journey?. Patient Educ. Couns..

[52] Vohra Y., Brown C.M., Moczygemba L.R., Wilfong L. (2023). Evaluating the relationship between patient activation and health-related quality of life (HRQOL) in patients with pancreatic cancer (PwPC). Support. Care Cancer.

[53] Mesters I., van den Borne B., De Boer M., Pruyn J. (2001). Measuring information needs among cancer patients. Patient Educ. Couns..

[54] Kanu C., Brown C.M., Rascati K., Moczygemba L.R., Mackert M., Wilfong L. (2021). Are health literacy and patient activation related to health outcomes in breast cancer patients?. HLRP Health Lit. Res. Pract..

[55] Patel M.I., Agrawal M., Blayney D.W., Bundorf M.K., Milstein A. (2024). Long-term engagement of patients with advanced cancer: Results from the EPAC randomized clinical trial. JAMA Oncol..

[56] Patel M.I., Ramirez D., Agajanian R., Agajanian H., Bhattacharya J., Bundorf K.M. (2020). Lay health worker-led cancer symptom screening intervention and the effect on patient-reported satisfaction, health status, health care use, and total costs: Results from a tri-part collaboration. JCO Oncol. Pract..

[57] Patel M.I., Smith K., Khateeb S., Park D.J. (2021). The effect of a lay health worker intervention on acute care use, patient experiences and end-of-life care: Results from a randomized clinical trial. J. Clin. Oncol..

[58] Adelson K., Rocque G. (2024). Community Health Worker Navigation for Patients with Cancer: It Is Time to Scale up. J. Clin. Oncol..

[59] Knowles M., Crowley A.P., Vasan A., Kangovi S. (2023). Community health worker integration with and effectiveness in health care and public health in the United States. Annu. Rev. Public Health.

[60] Patel M.I., Kapphahn K., Dewland M., Aguilar V., Sanchez B., Sisay E., Murillo A., Smith K., Park D.J. (2022). Effect of a Community Health Worker Intervention on Acute Care Use, Advance Care Planning, and Patient-Reported Outcomes Among Adults with Advanced Stages of Cancer: A Randomized Clinical Trial. JAMA Oncol..

[61] Patel M.I., Sundaram V., Desai M., Periyakoil V.S., Kahn J.S., Bhattacharya J., Asch S.M., Milstein A., Bundorf M.K. (2018). Effect of a Lay Health Worker Intervention on Goals-of-Care Documentation and on Health Care Use, Costs, and Satisfaction Among Patients with Cancer: A Randomized Clinical Trial. JAMA Oncol..

[62] Josfeld L., Keinki C., Pammer C., Zomorodbakhsch B., Hübner J. (2021). Cancer patients’ perspective on shared decision-making and decision aids in oncology. J. Cancer Res. Clin. Oncol..

[63] Kuosmanen L., Hupli M., Ahtiluoto S., Haavisto E. (2021). Patient participation in shared decision-making in palliative care–an integrative review. J. Clin. Nurs..

[64] Birkeland S., Bismark M., Barry M.J., Möller S. (2022). Is greater patient involvement associated with higher satisfaction? Experimental evidence from a vignette survey. BMJ Qual. Saf..

[65] Siebinga V.Y., Driever E.M., Stiggelbout A.M., Brand P.L. (2022). Shared decision making, patient-centered communication and patient satisfaction–A cross-sectional analysis. Patient Educ. Couns..

[66] Dimarco R., Guinigundo A.S., Valdueza C. (2023). Uncovering and Addressing Implicit Bias in Oncology. J. Adv. Pract. Oncol..

[67] Diaz D.A., Suneja G., Jagsi R., Barry P., Thomas C.R., Deville C., Winkfield K., Siker M., Bott-Kothari T. (2021). Mitigating Implicit Bias in Radiation Oncology. Adv. Radiat. Oncol..

[68] Lau-Min K.S., Guerra C.E., Nathanson K.L., Bekelman J.E. (2021). From Race-Based to Precision Oncology: Leveraging Behavioral Economics and the Electronic Health Record to Advance Health Equity in Cancer Care. JCO Precis. Oncol..

